# Efficacy of DAIR in managing Candida parapsilosis-infected total knee arthroplasty with five-year follow-up: A case report and review of literature

**DOI:** 10.1097/MD.0000000000036246

**Published:** 2023-11-24

**Authors:** Menglong Li, Mingrui Fan, Yuchen Zhang, Jianlin Xiao, Tong Liu, Qingwei Yu

**Affiliations:** a Department of Orthopedics, China-Japan Union Hospital of Jilin University, Changchun, China.

**Keywords:** Candida parapsilosis, continuous closed irrigation, DAIR, fungal periprosthetic infection, TKA

## Abstract

**Rationale:**

Fungal periprosthetic joint infections (fPJIs) are relatively uncommon, accounting for approximately 1% of all PJIs. Revision surgery is typically recommended for fungal infections; however, the physical and financial impact on patients is significant. In this report, we present a case of fPJI successfully treated with debridement, antibiotics, and implant retention (DAIR) with a favorable outcome over a 5-year period.

**Patient Concern:**

A 56-year-old male patient presented with a non-healing surgical incision 1 week after undergoing primary total knee arthroplasty on the right side.

**Diagnosis:**

Microbiological culture of the wound effusion identified Candida parapsilosis. Postoperatively, the patient exhibited a significant decrease in serum albumin levels and poor glycemic control. Both C-reactive protein and erythrocyte sedimentation rate were elevated.

**Interventions:**

A comprehensive DAIR procedure was performed, along with continuous closed irrigation using fluconazole for 1 week. The patient received intravenous voriconazole for 4 weeks, followed by oral fluconazole for an additional 3 months.

**Outcomes:**

At 1- and 5-year follow-up appointments, the patient C-reactive protein and erythrocyte sedimentation rate levels were within normal limits, and there was no evidence of swelling, erythema, or tenderness in the right knee joint, indicating no signs of infection.

**Lessons:**

DAIR is an effective treatment for early fPJIs, and continuous closed irrigation may provide specific advantages. The patient nutritional status plays a crucial role in the management of periprosthetic infections.

## 1. Introduction

Periprosthetic joint infections (PJIs) represent significant challenges and are prevalent complications following arthroplasty, with an incidence rate of up to 2% in total joint arthroplasties.^[1,2]^ Bacteria, particularly *Staphylococcus* species, are the primary cause of PJIs, while fungal PJIs (fPJIs) are considerably rarer, constituting approximately 1% of all PJIs.^[3,4]^ Candida species are the most common fungal pathogens involved.^[3,5]^ In contrast to bacterial PJIs, fPJIs typically present with more subtle clinical manifestations and are less likely to exhibit acute onset of symptoms. Consequently, diagnosis is frequently delayed, leading to postponed initiation of appropriate treatment.^[6]^ Fungal PJIs are notorious for their higher failure rates compared to bacterial infections.^[7]^ Currently, there are no established guidelines for fPJI management, with treatment options ranging from antifungal drugs and debridement to implant retention and 1- or 2-stage revision strategies, all of which yield variable outcomes.^[8–11]^ The 2-stage approach is widely recognized as the concrete treatment method.^[12,13]^ On the other hand, debridement, antibiotics, and implant retention (DAIR) is a feasible treatment strategy for acute prosthetic joint infection (PJI).^[14,15]^ The technique aims to maintain a well-fixed implant by reducing the intraarticular microbial load through surgical irrigation and debridement of affected tissues, followed by antibiotic therapy. Although success rates vary significantly (33%–88%),^[16–21]^ DAIR offers several advantages over revision prosthetic surgery, such as reduced morbidity rates, faster postoperative recovery, and lower associated economic costs.

This case report emphasizes a successful example of primary prosthesis-sparing debridement for a fungal periprosthetic infection following total knee replacement, with a 5-year follow-up. The patient underwent multiple arthrocentesis procedures, and exudate cultures identified Candida parapsilosis as the causative agent. Taking into account the patient physical condition and the acute onset of infection, the medical team opted for prosthetic debridement retention instead of pursuing a revision surgery.

## 2. Case presentation

**Figure 1.**
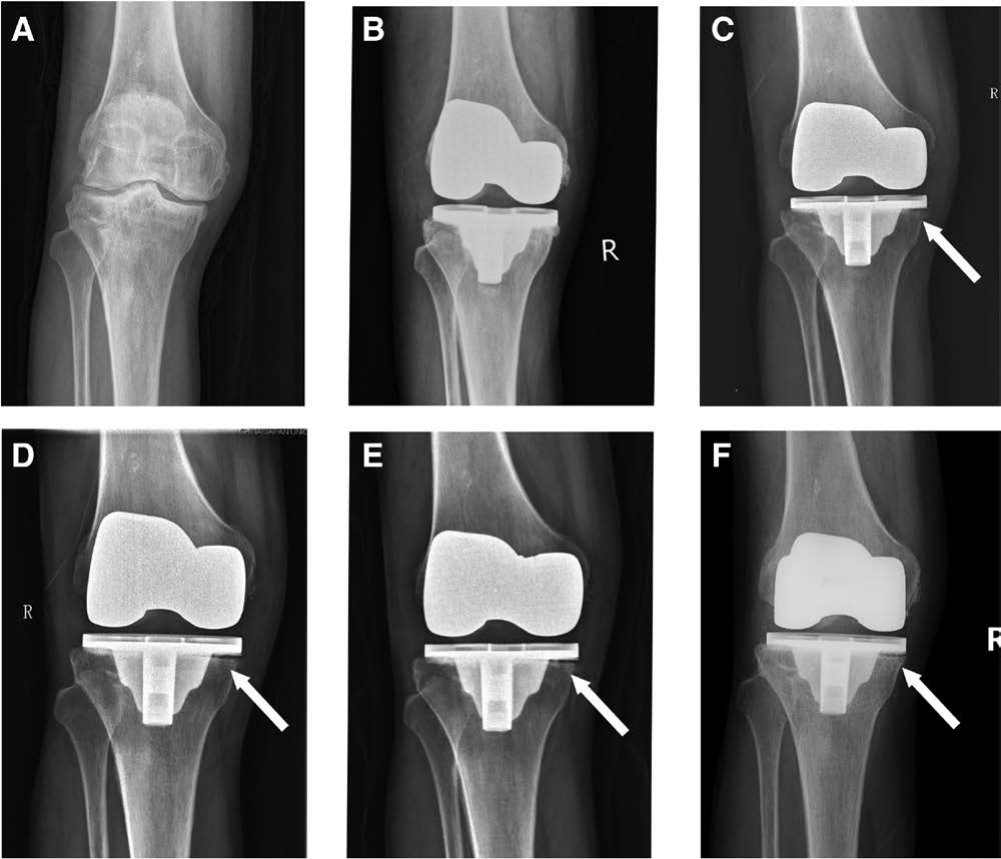
(A–F) AP radiographic progression of the right knee: (A) Preoperative AP radiograph demonstrating severe osteoarthritis; (B) postoperative radiograph following primary total knee arthroplasty; (C) AP radiograph taken 2 mo after DAIR, showing a radiolucent line beneath the medial tibial tray; (D) at the 5-mo follow-up, the radiolucent line has progressed; (E) at the 1-yr follow-up, the radiolucent line remains unchanged; (F) at the 5-yr follow-up, new bone formation is observed below the tibial component. AP = anteroposterior, DAIR = debridement, antibiotics, and implant retention.

This study was approved by the Ethics Committee of the China-Japan Union Hospital of Jilin University. Signed informed consent was obtained from the patient in accordance with the Declaration of Helsinki. A 56-year-old male patient underwent a total knee arthroplasty (TKA) on the right side due to osteoarthritis (OA) of the knee (Fig. 1A). His medical history includes type 2 diabetes mellitus for over 10 years (managed with insulin), and a 40-year history of smoking for 1 pack per day. Upon admission, his fasting blood glucose level was 6.59 mmol/L, and all serum infective indicators were within the normal range. A routine TKA procedure was performed on March 13, 2017. A postoperative standard anteroposterior (AP) radiograph was taken (Fig. 1B), and cephalosporin was administered intravenously for infection prevention for 3 days post-operation. The medical team monitored the patient blood glucose level 7 times per day after surgery (including before and after 3 meals and at bedtime), revealing blood glucose levels as high as 10 mmol/L before meals and 17 mmol/L after meals. Aware of the risks of diabetes-related complications, the patient intentionally reduced his dietary intake, resulting in poor post-operative nutritional intake and a significant decrease in his serum albumin level (33.3g/L, compared to 43.3g/L preoperatively). Seven days after surgery, a 2-cm-long poorly healed wound in the upper part of the TKA incision was observed, and effusion was collected for microbiological culture. Three days later, the patient requested discharge. That night, the patient temperature rose to 39.7°C, and the medical team advised the patient to return to the hospital as soon as possible, as the result of the microbiological culture indicated Candida parapsilosis.

**Figure 2.**
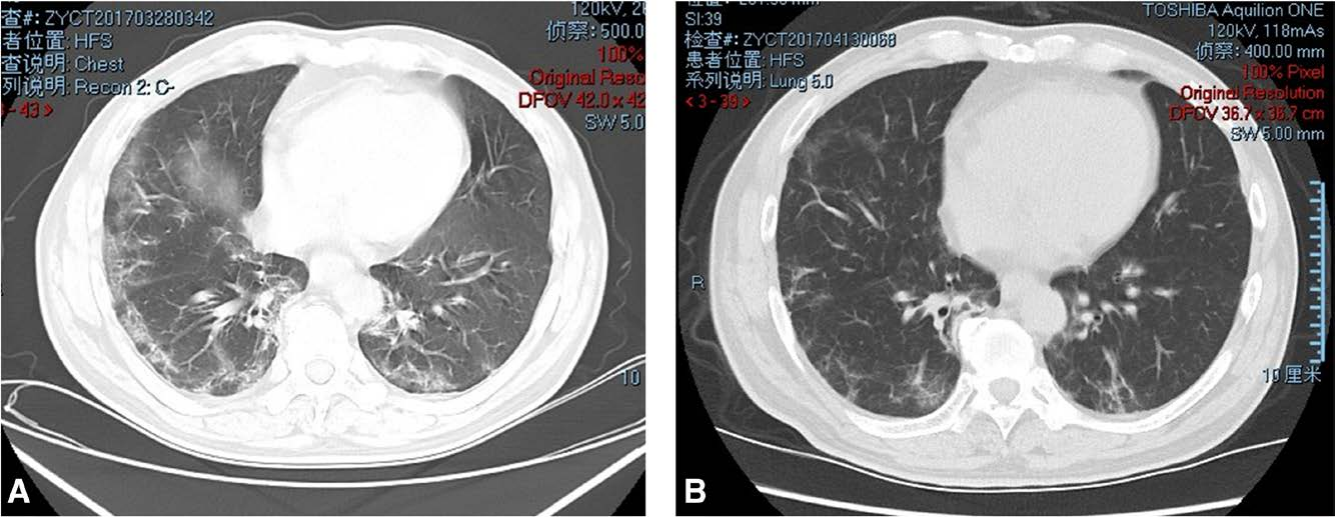
(A and B) Radiologic progression of interstitial pneumonia: (A) Diagnosis of interstitial pneumonia 2 wk following primary total knee arthroplasty; (B) chest CT scan demonstrating resolution of pneumonia during the 2nd wk of antibiotic treatment. CT = computed tomography.

On March 28, the patient was admitted to the Respiratory Department with a 4-day history of fever accompanied by cough and sputum production. His partial pressure of oxygen was 60 mm Hg in arterial blood. An emergency chest computed tomography was performed, confirming a diagnosis of interstitial pneumonia (Fig. 2A). The patient physical examination revealed a 3 cm open wound in the upper part of the right knee joint, where the skin, subcutaneous tissue, and fascia were poorly healed with a small amount of exudate. Sputum culture revealed Streptococcus viridans, and treatments included antibiotics, airway diastolic medication, expectorant, oxygenation, and supportive care. Another culture of the right knee aspiration was taken, which again indicated Candida parapsilosis infection. On April 1, 2017, the patient respiratory infection was controlled, and he was transferred to the Orthopaedic Department for further treatment of his right knee. His C-reactive protein (CRP) level was 59.4 mg/L, erythrocyte sedimentation rate (ESR) was 70 mm, and fasting blood glucose level was 7.2 mmol/L (well managed by an endocrinologist). Based on laboratory exams, culture results, and clinical presentations, a diagnosis of fPJI was made. Considering the patient poor nutritional status and the fact that both interstitial pneumonia and fungal infection are primarily conditional infections, enhanced protein support was initiated. Since the fPJI had an acute onset and the patient and his family refused revision surgeries, a DAIR procedure was performed. Intraoperatively, the components were mechanically stable, and all necrotic and proliferated tissues were thoroughly removed. The gutters, backside of the capsule, and synovial tissues was deeply debrided after removing the insert. The surfaces of the prosthesis were brushed and irrigated multiple times with hydrogen peroxide, povidone-iodine, isotonic sodium chloride solution, and fluconazole. Finally, a new insert was replaced, and an intraarticular dual tube closed irrigation system was inserted.

Postoperatively, a solution containing 1000 mL isotonic sodium chloride and 100 mg fluconazole was used for continuous irrigation 4 times per day for 1 week using the closed irrigation system. Intravenous Voriconazole (400 mg/day) was admitted for 4 weeks during hospitalization without adverse events, and intravenous Piperacillin-tazobactam (9 g/day) was used to treat interstitial pneumonia for 3 weeks. A chest computed tomography performed during the 2nd week of antibiotic treatment showed remission of the pneumonia (Fig. 2B). The patient was discharged 1 month after the DAIR procedure, with a well-healed incision and reduced CRP and ESR levels (46.0 mg/L and 46mm, respectively), as well as a partial pressure of oxygen of 79 mmHg. Oral Fluconazole (200 mg/day) was prescribed for 3 months. At the 2-month follow-up, CRP and ESR levels were further reduced to 3.9 mg/L and 30 mm. However, an AP radiograph of the right knee revealed a radiolucent line below the medial tibial component (Fig. 1C). Since the patient did not report any pain or sign of component loosening, close follow-ups were scheduled. On September 20, 2017 (five months after DAIR), the translucent line had progressed (Fig. 1D), but there was no swelling, redness, or tenderness in the right knee joint, and no restriction in standing or walking. At the 1-year follow-up, the patient CRP and ESR levels were within normal limits, and the AP radiograph showed that the translucent line below the tibial tray was similar to the previous exam (Fig. 1E). At the 5-year follow-up, new bone formation was observed below the tibial component on X-ray (Fig. 1F), with a range of motion of the right knee at 0 to 110 degrees and no sign of infection.

## 3. Review of literature

### 3.1. Etiology

Most patients with PJI present 1 or more specific risk factors, such as immunosuppression, obesity, diabetes, multiple previous revision surgeries, long-term antibiotic treatments, or even surgical season.^[22–27]^ Recent findings indicate that nutritional status, particularly albumin levels, plays a crucial role in PJI management. Hypoalbuminemia may contribute to increased failure rates in treatment outcomes.^[28–30]^ Furthermore, the majority of PJI cases are associated with bacterial infections, predominantly involving *Staphylococcus* species. Fungal PJI is considerably rarer, accounting for approximately 1% of the total number of PJI cases.^[3,4]^ Consequently, typical cases of fungal PJI are less frequently encountered, and the clinical presentation of fungal PJI tends to be more subtle, with less acute symptoms.^[6]^ The most common pathogen implicated in fungal PJI is Candida species.^[3,5]^ Other types of fungi causing PJI are seldom reported. Preoperative synovial fluid culture should not be employed as a screening test in the diagnosis of PJI. Even a negative synovial culture does not provide the surgeon with certainty that the joint is sterile.^[31]^

### 3.2. Diagnosis

Currently, there is no gold standard or widely accepted criteria for the specific diagnosis of fPJI. The 2018 Musculoskeletal Infection Society criteria for PJI in hip and knee are commonly used by surgeons when addressing fPJI.^[32]^ However, it is evident that fPJI presents with different clinical manifestations compared to bacterial infections. Most cases exhibit subtle inflammatory symptoms,^[33]^ while only a few present as acute infections that can be diagnosed in the early stage.^[34–39]^ Initial diagnostic tools for fPJI include ESR and CRP, as serum inflammatory biomarkers are easily accessible worldwide, cost-effective, and can be routinely performed preoperatively. These markers can serve as the first indicators for PJI in the absence of clinical signs. A review of commonly used serum markers indicates that CRP and fibrinogen have the highest diagnostic accuracy.^[40]^ However, according to the medical histories of most reported cases in the literature, these parameters demonstrate low accuracy in confirming low-grade infections,^[40–43]^ which are more common in fungal PJI. As a result, serum inflammatory markers should be considered suggestive rather than definitive criteria. When CRP levels are elevated, clinicians should further investigate the possibility of PJI. Normal levels of serum inflammatory markers do not exclude infection.^[40–42]^ Synovial fluid culturing provides a definitive diagnosis in fPJI.^[44]^ Reported cases in the literature typically have clear culture results identifying specific pathogens, but higher percentages of fungal infection in all PJI cases can be expected in reality, as culturing fungi can be challenging. Culture-negative cases that respond poorly to antibiotics may be due to such situations.

### 3.3. Classification and treatment

PJI can generally be classified into early infection (less than 4 weeks) and late infection (more than 4 weeks).^[45]^ The primary treatment options for PJI include DAIR, as well as 1-stage revision, and 2-stage revision.^[46]^ Early infections can be addressed through aggressive debridement, replacement of modular components, and retention of fixed components, while late infections typically necessitate removal of the prosthetic component.^[47]^ In addition to the duration of infection, the patient systemic status and local soft tissue conditions should also be considered.^[48]^ Many researchers believe that 1- or 2-stage revisions are required in most fPJI cases, as the majority of these cases present mild clinical symptoms and are often diagnosed late.^[26]^ One-stage revision surgery offers the advantages of requiring only 1 operation, reducing the duration of antibiotic therapy, and decreasing the risk of liver and kidney failure, hospitalization time, and financial costs. Conversely, 2-stage revision is considered the “gold standard” for PJI treatment, providing the surgeon with a sufficient interval to achieve an adequate antimicrobial treatment cycle for the patient.

Despite the variable success rates of DAIR, some surgeons prefer this technique in fPJI management due to its faster postoperative recovery, lower economic costs, and simpler operation. A summary of fPJI cases treated with DAIR or similar procedures was listed in Table 1.^[3, 5, 38, 39, 49–54]^ The primary goal of DAIR is to preserve a well-fixed prosthesis and control periprosthetic infection through continuous antibiotic flushes and 1 or more debridement. However, only patients with early PJI should be considered for this treatment, and it should not be used in patients with risk factors for persistent or recurrent infection. Cases with poor soft tissue envelopes, immunocompromised conditions, or drug-resistant pathogens should be excluded.^[55]^ According to our review, early detection of fungal infection may still be an indication for DAIR.

**Table 1:** A summary of fPJI cases treated with DAIR or similar procedures in the literature.

Author	Yr	Number of cases	Age at diagnosis, yr	Organism	Site	Time from primary procedure	Treatment	Medication	Follow-up	Outcome
Fukasawa et al^[39]^	1997	1	80	Candida parapsilosis, Pseudomonas aeruginosa	Knee	2 mo	Continues closed suction-irrigation	Fluconazole (irrigation), Dibekacin (intraarticular injection), Ceftazidime + clindamycin (iv), Fluconazole + norfloxacin (oral)	2 yr	cured
Zhu et al^[38]^	2014	1	44	Candida glabrata	Hip	2 mo	DAIR	amphotericin B+ voriconazole (iv)	3 mo	Cured
Dutronc et al^[49]^	2010	1	85	Candida albicans	Hip	Immediate	Debridement and prosthetic retention	Fluconazole + 5-fluorocytosine	NR	Cured
Hwang et al^[5]^	2012	4	69 (mean)	Candida species	Knee	19.6 mo (mean)	DAIR	Amphotericin B + fluconazole	4.3 yr (mean)	Removal of the components
Azzam et al^[3]^	2009	7	64 (mean)	Candida species	Hip/knee	25 mo (mean)	DAIR	Fluconazole or Amphotericin B	45 mo (mean)	Removal of the prosthesis (5 cases), suppressed with oral fluconazole (2 cases)
Bland et al^[50]^	2009	1	55	Candida albicans	Knee	1 mo	DAIR	Micafungin + fluconazole	2 mo	Removal of the prosthetic knee hardware
Brown et al^[51]^	2018	1	84	Candida albicans	Hip	NR	DAIR	NR	NR	Reinfection
		1	37	Candida albicans	Hip	NR	DAIR	NR	NR	Revision
		1	76	Candida albicans	Hip	NR	DAIR	NR	NR	Revision
		1	71	Candida parapsilosis	Knee	NR	DAIR	NR	NR	Reinfection
Fowler et al^[52]^	1998	1	84	Histoplasma capsulatum	Hip	8 yr	DAIR	Itraconazole	3 yr	Suppressed
Wada et al^[53]^	1998	1	77	Candida parapsilosis	Knee	3 wk	Curettage and continuous irrigation	Fluconazole (irrigation, oral)	3 yr	Cured
Brooks et al^[54]^	1998	1	64	Candida parapsilosis	Knee	1.5 yr	DAIR	Amphotericin B + fluconazole	2 yr	Cured

DAIR = debridement, antibiotics, and implant retention, fPJI = fungal prosthetic joint infection, iv = intravenous, NR = not reported.

The continuous closed irrigation technique, traditionally applied in general or neurosurgery settings, can theoretically introduce topical high-dose antibiotics and eliminate debris in the affected joints of PJI cases.^[56]^ Some surgeons have used this technique in knees with septic arthritis^[57,58]^ and a few cases involving infected total knee components.^[59,60]^ However, the effectiveness of this technique in PJI management remains uncertain.^[56]^ In our reviewed fPJI cases treated with DAIR, 2 cases underwent continuous irrigation, both achieving clinical cure. Although the number of successful cases is too small to draw definitive conclusions, continuous closed irrigation may be a potential direction for managing fungal prosthetic infections considering the low cure rate of DAIR in treating fPJI.

## 4. Discussion

Understanding risk factors is crucial for predicting and managing PJI. In a study based on an arthroplasty database, Perni et al^[61]^ observed that the most common comorbidity in their cohort was OA, followed by hypertension. Other factors such as rheumatoid arthritis, chronic kidney disease, diabetes mellitus (type 1 or 2), anemia, and ischemic heart disease were also associated with PJI development. The prevalence of diabetes mellitus, chronic kidney disease, OA, and rheumatoid arthritis was statistically different in PJI patients compared to the general population. Diabetes was significantly associated with an increased incidence of PJI,^[62–65]^ as poor glycemic control is often linked to higher infection rates. In the present case, the patient had a long history of diabetes mellitus, and poor postoperative glycemic control further increased his risk of infection. Additionally, the patient nutritional status was unsatisfactory due to excessive dietary restriction for glycemic control, leading to a significant decrease in albumin levels compared to admission. Hypoalbuminemia is known to be associated with poor PJI outcomes.^[28–30]^ Furthermore, the development and prognosis of respiratory diseases, such as the postoperative interstitial pneumonia in this case, have been found to be closely related to nutritional status.^[66]^ Therefore, enhanced protein support was considered crucial in managing the patient overall condition. Ultimately, we believe that systematic glucose management and proper nutritional supplementation played important roles in achieving a favorable outcome in this case.

Candida species are the most common fungi found in fPJI cases, with Candida parapsilosis identified in the current case accounting for approximately 27% of all cases in the literature.^[26]^ Fortunately, the microorganism in this case is easier to manage compared to Candida albicans, which can form complex biofilms.^[67,68]^ Brooks et al^[54]^ reported a total knee case infected with Candida parapsilosis that was ultimately cured with a DAIR procedure despite being categorized as a late infection. Inflammatory markers such as ESR and CRP are believed to have limited ability in the early diagnosis of fPJI, as they often show minimal elevation in most cases.^[43,69,70]^ In this case, ESR and CRP levels were both significantly increased at the onset of infection and gradually decreased when treatment was effective; however, these markers may have been positively influenced by the patient concurrent respiratory infection. As evidenced in the literature review, many fPJI cases present mild symptoms and comparatively low inflammatory reactions, leading to delayed diagnoses. In contrast, acute onset fungal infections may exhibit clinical presentations and laboratory results similar to bacterial PJIs.

In this case, the patient PJI was an early infection (less than 4 weeks) and was successfully treated with DAIR; however, the more robust and widely accepted approach remains 2-stage revision surgery.^[26]^ The current literature does not strongly support retained prosthesis debridement for fPJI treatment, primarily due to its indolent performance that often results in late diagnosis.^[26]^ Acute onset of infection is comparatively rare, accounting for only 5 out of 18 DAIR cases with reported time intervals. However, an 80% success rate was associated with fPJI cases detected within 2 months of primary surgery and treated with DAIR (Table 1), suggesting that patients with early fungal infections may still be good candidates for prosthesis retention. Another reason for choosing the current strategy was understanding the patient condition and developing a personalized treatment plan tailored to the patient unique circumstances and needs. The patient had comorbidities including hypoalbuminemia and poorly controlled diabetes, and interstitial pneumonia and respiratory failure increased the likelihood of systemic complications for revisions. The patient overall status is critical in making surgical decisions in PJI management.^[48]^ Continuous closed irrigation provided steady high-dose local medication infiltration in a manageable functional period.^[56]^ Although it has been found to have fewer advantages over DAIR alone in bacterial infection,^[56]^ continuous closed irrigation may be an option for treating fPJI considering the high hepatic burden of antifungal agents in systemic use. To date, 2 cases in the literature (Table 1) and our patient with fPJI have been successfully treated with this technique, and we anticipate further evidence and research on this topic.

## 5. Conclusions

fPJI is a rare occurrence, and its clinical presentations are often subtle, which can result in delayed diagnosis and necessitate revisions in most cases. In this manuscript, we describe an early fungal PJI case that was successfully treated using DAIR, and continuous closed irrigation. Additionally, we reviewed the applications of DAIR in fPJI treatment. We believe that the patient nutritional status plays a critical role in the management of fPJI. For early periprosthetic fungal infections, DAIR can still be a recommended option, and continuous closed irrigation may offer specific benefits in fPJI management. Further evidence and research are needed to confirm our findings.

## Author contributions

**Conceptualization:** Jianlin Xiao, Tong Liu.

**Data curation:** Menglong Li, Mingrui Fan, Yuchen Zhang.

**Formal analysis:** Menglong Li, Mingrui Fan.

**Funding acquisition:** Tong Liu.

**Investigation:** Menglong Li, Mingrui Fan, Yuchen Zhang.

**Resources:** Tong Liu.

**Supervision:** Jianlin Xiao, Tong Liu, Qingwei Yu.

**Writing – original draft:** Menglong Li, Mingrui Fan.
